# Automatic Schizophrenia Detection Using Multimodality Media via a Text Reading Task

**DOI:** 10.3389/fnins.2022.933049

**Published:** 2022-07-14

**Authors:** Jing Zhang, Hui Yang, Wen Li, Yuanyuan Li, Jing Qin, Ling He

**Affiliations:** ^1^College of Biomedical Engineering, Sichuan University, Chengdu, China; ^2^Mental Health Center, West China Hospital of Sichuan University, Chengdu, China; ^3^Centre for Smart Health, School of Nursing, The Hong Kong Polytechnic University, Hong Kong, Hong Kong SAR, China

**Keywords:** schizophrenia, reading deficit, multimodality, speech, video, head movement, reading fluency

## Abstract

Schizophrenia is a crippling chronic mental disease that affects people worldwide. In this work, an automatic schizophrenia detection algorithm is proposed based on the reading deficit of schizophrenic patients. From speech and video modalities, the automatic schizophrenia detection algorithm illustrates abnormal speech, head movement, and reading fluency during the reading task. In the speech modality, an acoustic model of speech emotional flatness in schizophrenia is established to reflect the emotional expression flatness of schizophrenic speech from the perspective of speech production and perception. In the video modality, the head-movement-related features are proposed to illustrate the spontaneous head movement caused by repeated reading and unconscious movement, and the reading-fluency-related features are proposed to convey the damaged degree of schizophrenic patients' reading fluency. The experimental data of this work are 160 segments of speech and video data recorded by 40 participants (20 schizophrenic patients and 20 normal controls). Combined with support vector machines and random forest, the accuracy of the proposed acoustic model, the head-movement-related features, and the reading-fluency-related features range from 94.38 to 96.50%, 73.38 to 83.38%, and 79.50 to 83.63%, respectively. The average accuracy of the proposed automatic schizophrenia detection algorithm reaches 97.50%. The experimental results indicate the effectiveness of the proposed automatic detection algorithm as an auxiliary diagnostic method for schizophrenia.

## Introduction

Schizophrenia is a disabling disease worldwide, which is accompanied by speech or behavior disorders (Patel et al., 2014). Schizophrenia not only creates social functional defects in patients but also affects their physical health (Dickerson et al., 2014; Silverstein and Rosen, 2015; Schulz et al., 2020). The early and accurate diagnosis and treatment of schizophrenia have long-term advantages for patients (Soaresweiser et al., 2015). Currently, schizophrenia diagnosis is based on the standardized scales developed by authorities (ICD-10, ICD-11, DSM-5, etc.) (Wawer et al., 2021). In the process of clinical diagnosis, the subjective personal experience of doctors is added to the diagnosis (Cruz-Martinez et al., 2022). Objective and effective biological indicators would help doctors with auxiliary diagnoses.

Clinical studies have shown that schizophrenic patients have dyslexia in reading. Researchers found that schizophrenic patients have a dyslexia-related risk gene DCDC2 (doublecortin domain-containing protein 2) (Jamadar et al., 2011), and their volume of brain regions related to reading is significantly decreased (Leonard et al., 2008; Stefansson et al., 2013). Schizophrenic patients perform significantly worse on single-word reading tasks than normal controls (Revheim et al., 2014). In connected text reading tasks, they also show defects in reading speed and comprehension ability (Revheim et al., 2014; Whitford et al., 2018). Studies have shown that defects in reading tasks can be used as one of the biological markers for diagnosing schizophrenia.

Previous studies have shown that the reading defects of schizophrenic patients can be reflected in their abnormal speech, head movement, and reading fluency.

1) Abnormal speech of schizophrenia: Clinical research proves that the brain nerve activation pattern involved in speech processing in schizophrenic patients differs from normal controls in reading tasks (Angrilli et al., 2009). The decrease in saccade amplitude in schizophrenic patients during reading is related to decreased speech processing ability (Whitford et al., 2013). Compared with normal controls, schizophrenic patients have a smaller emotional range in the reading task (Docherty et al., 2012), which is reflected in speech as smaller variations in pitch and intensity (Compton et al., 2018; Meyer et al., 2021). Studies have extracted the relevant features of pitch and intensity to reflect the emotional variations in schizophrenic speech: Compton et al. (2018) used the standard deviation of *F*_0_ to represent the pitch variations in speech and calculated an average of the average SDs of intensity changes over a 20-s window to reflect the intensity variations in schizophrenia. They only carried out statistical analysis on the extracted features, which were not further used for automatically classifying schizophrenia. Chakraborty et al. (2018) extracted the *F*_0_, *F*_0_ envelope, and the intensity features using the speech analyzer to reflect the speech pitch and intensity variation in schizophrenia, and achieved the automatic diagnosis of schizophrenia with 79–86%. Tahir et al. (2019) calculated the speech envelope's minimum, maximum, and mean values to represent the intensity of schizophrenic speech, and the accuracy of their automatic classification of schizophrenia was 59.3%.

2) Abnormal head movement of schizophrenia: The reading process is accompanied by body movements. Motor processing and speed discrimination are needed in the body movement process. Clinical experiments have shown that the activation of the V5/MT (Visual area 5/Middle Temporal) region decreases during motor processing and speed discrimination in patients with schizophrenia (Chen et al., 2008). The lower activation level of the V5/MT region is related to a higher speed discrimination threshold and leads to abnormal body movement (Demb et al., 1998). Head movement is mainly body movement when reading the specified text in a fixed position. Studies have reported that schizophrenic patients have significant differences in head movement rates from controls (Abbas et al., 2021), and produce unconscious and unnecessary head movement in visual tasks (Yoo et al., 2005). The head movement measuring methods used in the present research include motion energy analysis (MEA) (Kupper et al., 2010, 2015) and head-mounted motion sensors (Leask et al., 2012). MEA reflects the subject's head movement through the gray changes between the recorded video frames, which quantifies the amount of movement but is blind to the movement trajectory. The head-mounted motion sensors are in direct contact with subjects, which create an additional psychological burden on schizophrenic patients.

3) Abnormal reading fluency of schizophrenia: Reading fluency deficiency is one of the apparent characteristics of schizophrenic patients in reading tasks (Revheim et al., 2014; Whitford et al., 2018). Clinical experiments have found that during the verbal fluency task, the left upper temporal cortex of schizophrenic patients fails to show normal blood flow reduction, which leads to abnormal fluency (Frith et al., 1995). In addition, negative symptoms and attention disorder are also factors affecting speech fluency in patients with schizophrenia (Brébion et al., 2013). Badcock et al. (2011) found through experiments that schizophrenic patients have defects in the fluency of expressing verbs and nouns. Rossell (2006) found that schizophrenic patients showed fluency defects in different emotional expression tasks. Other studies have also proven fluency defects in schizophrenic patients compared with normal controls through verbal fluency tests (Rinaldi et al., 2013; Krukow et al., 2017). Most of the existing fluency-related studies evaluated the subjects' fluency by the number of words correctly expressed in a fixed time, which ignore the fluency defect information in the continuous expression process.

In this work, based on the abnormal performance of schizophrenic patients in reading tasks, an automatic schizophrenia detection algorithm is proposed from the speech and video modalities. In the speech modality, based on the abnormal emotional expression of schizophrenic patients from the perspective of speech production and perception, the acoustic model of speech emotion expression is established. In the video modality, the head-movement-related features are proposed based on the mapping from three-dimensional head movement to two-dimensional images, which quantify the subjects' spontaneous head movements in the reading task. The reading-fluency-related features (RRFs) are proposed based on the information extracted by transfer learning from the time and frequency domains of the mouth movement sequences.

The main contributions of this work are summarized as follows:

(a) A new automatic schizophrenia detection algorithm is proposed from multimodality based on the abnormal performance of schizophrenic patients in the reading task.
(b) An acoustic model of speech emotional flatness in schizophrenia is established to reflect the emotional flatness of speech expression in patients with schizophrenia from the perspective of speech production and perception.
(c) The head-movement-related features are proposed from the video modality recorded during the reading task. Based on the mapping from three-dimensional head movement to two-dimensional images, the head-movement-related features are proposed to reflect the spontaneous head movement caused by repeated reading and unconscious movement.
(d) The RRFs are proposed from the mouth movement during the reading task. Based on the ResNet101 pretrained model, the feature extraction network is built by using transfer learning, and the RRFs are proposed to reflect the degree of reading fluency of subjects.

## Materials and Methods

### Dataset

The data from this experiment were recorded from 20 schizophrenic patients (11 females and 9 males) and 20 normal controls (15 females and 5 males). The subjects who participated in the recording came from the Psychiatry Department of the Mental Health Center, Sichuan University, which is one of the four regional centers of psychiatry in China. The controls had no mental history or family genetic history of psychosis and no other mental health problems. All participants were informed and voluntarily participated in the experiment.

Due to the difficulty of data collection of schizophrenic patients, the number of patient samples in the study of automatic detection of schizophrenia is relatively small. Ethical review, signing of informed consent, and other preparations are required before pathological data collection. After data collection, some collected data may not meet the inclusion criteria of the pathological multimodal database and need to be eliminated. In addition, the selection of the control group needs to be matched according to the general information of the patients (including age, gender, and education level), which is also difficult. The whole process of data acquisition is long and difficult, and the effective data are limited.

In recent years, in the research on the analysis of abnormal speech (Holmlund et al., 2020; Tang et al., 2021) and head movement (Robson et al., 2016; Abbas et al., 2021) of schizophrenia, the sample number of schizophrenic patients is about 20: Tang et al. (2021) use the dataset of 20 patients vs. 11 controls; Holmlund et al. (2020) use the dataset of 25 patients vs. 79 controls; Abbas et al. (2021) use the dataset of 18 patients vs. 9 controls; Robson et al. (2016) use the dataset of 23 patients vs. 23 controls.

The experimental data of this work are 160 segments of speech and video data recorded by 40 participants, which contain 80 segments of data from schizophrenic patients and 80 segments of data from normal controls. During recording, the subjects were asked to sit in front of the recording screen and read four text segments. The reading texts used in this experiment were formulated by the specialist psychiatrist of the Psychiatry Department of the Mental Health Center, Sichuan University, according to the DSM-5 standard, which does not contain rare words and words that are difficult to understand. The educational level of the subjects met the reading needs of the experimental texts, and all subjects read for the first time. The video recording equipment for this experiment was a Logitech C922 pro with 1920 ^*^ 1080 resolution and 30 fps frame rate. The sampling frequency of the corresponding audio is 44,100 Hz.

### Automatic Schizophrenia Detection Algorithm Using Multimodality Media via a Text Reading Task

Previous studies have reported that schizophrenic patients show defects in reading tasks (Whitford et al., 2018; Mitelman et al., 2021). Clinical experiments proved that the reading defects of schizophrenia can be reflected in speech, head movements, and reading fluency in reading tasks (Demb et al., 1998; Chen, 2011; Whitford et al., 2013; Revheim et al., 2014).

**Figure 1:**
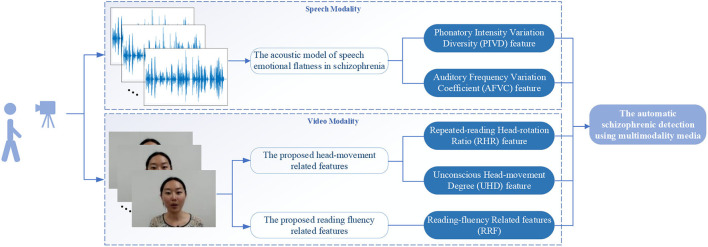
The flow diagram of the automatic schizophrenic detection algorithm using multimodality media *via* a text reading task.

In this work, an automatic schizophrenic detection algorithm based on the reading defects of schizophrenia is proposed from speech and video modalities (as shown in Figure 1). The calculation in each modality is introduced in subsections The Acoustic Model of Speech Emotional Flatness in Schizophrenia and The Proposed Head-Movement and Reading-Fluency-Related Features From the Video Modality, respectively.

#### The Acoustic Model of Speech Emotional Flatness in Schizophrenia

Speech disorder is recognized as one of the significant symptoms of schizophrenia (Meyer et al., 2021; Tan et al., 2021). Previous studies have shown that because of emotional flattening symptoms, schizophrenic patients have defects in speech expression, especially emotional expression (McGilloway et al., 2003; Alberto et al., 2019; Bora et al., 2019). Abnormal emotional expression is manifested in the production and perception of speech. From the perspective of speech production, the schizophrenic patients' vocal organs activity and vocal cord resonance amplitude decrease, which suppress the variation in speech intensity (Martínez-Sánchez et al., 2015; Compton et al., 2018). From the perspective of speech perception, the speech of schizophrenic patients has more minor pitch variations and lower pitch richness (Leitman et al., 2010; Henry et al., 2015).

**Figure 2:**
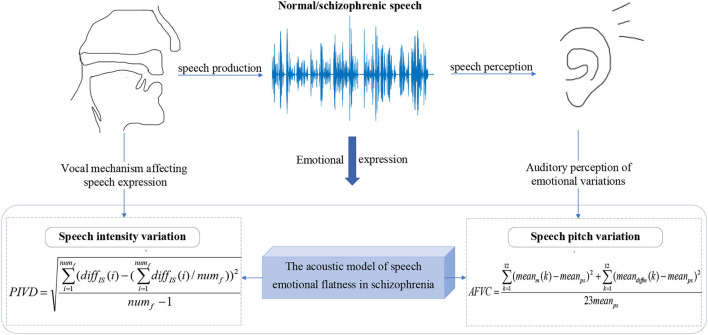
The diagram of the acoustic model of speech emotional flatness in schizophrenia.

In this work, an acoustic model of speech emotional flatness in schizophrenia is established to illustrate the emotional flattening symptoms of schizophrenia. As shown in Figure 2, from the perspective of speech production and perception, the phonatory intensity variation diversity (PIVD) feature and auditory frequency variation coefficient (AFVC) feature are proposed.

##### The Proposed PIVD Feature

Previous studies have shown that schizophrenic patients have defects in emotional expression, making it difficult to express their emotional variations correctly (Zou et al., 2018). From the perspective of speech production, the lower activity of vocal organs and the decreased amplitude of vocal cord resonance lead to the lack of variations in speech intensity of schizophrenic patients (Martínez-Sánchez et al., 2015; Compton et al., 2018). In addition, the abnormal brain discharge mechanism of self-generated speech in schizophrenic patients also affects the intensity variation required to express emotion in the reading process (Whitford et al., 2017; Ford et al., 2021).

In this work, PIVD is proposed to quantify the variations in speech intensity, which reflect the emotional variations in the subject from the perspective of speech production.

Considering that the speech signal in one frame can be regarded as stable, the average intensity of each frame is used to represent the overall intensity level of the speech frame. To quantify the speech intensity variation, the first-order difference of the intensity means sequence is calculated in this work. The first-order difference of the intensity means sequence represents the intensity difference between two adjacent frames and dynamically reflects the intensity variation in the speech with time.

To evaluate the diversity of subjects' speech intensity variations, PIVD is proposed to quantify the degree of the speech intensity variations. PIVD is the standard deviation of the extracted first-order difference sequence. The calculation formula of PIVD is as follows:


(1) $$PIVD\;=\;\sqrt{\frac{{\sum {}_{i=1}^{num_{f}}\left(diff_{IS}\left(i\right)-\left(\sum {}_{i=1}^{num_{f}}\frac{diff_{IS}\left(i\right)}{num_{f}}\right)\right)}^{2}}{num_{f}-1}}$$

(2) $$mean_{IS}\left(x\right)=\frac{\sum {}_{k=1}^{N}IS_{x}\left(k\right)}{len_{f}}$$

(3) $$\begin{array}{rcl} {diff}_{IS}\left(x\right) & = & {mean}_{IS}\left(x+1\right)-{mean}_{IS}\left(x\right) \end{array}$$

where *mean*_*IS*_ denotes the calculated average intensity of each frame, *IS*_*x*_ is the intensity sequence of the *x*th frame of the speech, *len*_*f*_ represents the frame length, *diff*_*IS*_ denotes the calculated first-order difference of the intensity means sequence, and *num*_*f*_ represents the number of frames of the speech.

PIVD represents the fluctuation in speech intensity variation caused by the coordination of vocal organs, which reflects the diversity of speech intensity variations.

##### The Proposed AFVC Feature

The speech-emotional flattening expression of schizophrenic patients is also reflected at the auditory level. From the perspective of speech perception, the poor emotional expression of schizophrenic patients is specifically manifested in lower pitch variations and richness (Leitman et al., 2010; Henry et al., 2015; Espinola et al., 2020; Maneval, 2021).

The pitch of the sound heard by the human ear is not linearly proportional to the real sound frequency, while the mel-frequency scale is more in line with the auditory characteristics of the human ear. Mel Frequency Cepstral Coefficient (MFCC) is extracted based on the mel-frequency scale, which is a speech frequency feature reflected from the perspective of hearing (Ali et al., 2021). The degree of MFCC variation can reflect the pitch variation in the auditory system.

To obtain the speech frequency characteristics perceived from the auditory angle, the MFCC parameters of each speech are extracted first. In this work, the MFCC parameters of each frame of speech are calculated by using 12 mel filters and the Hamming window function, which represent the frequency information on the 12 non-linear scales perceived by human ears.

The second-order differential MFCC parameters reflect the dynamic relationship of MFCC parameters between adjacent three frames, which supplement the static defect of MFCC. To further supplement the speech frequency information perceived by human ears, the second-order differential MFCC parameters (*diff*_*m*_) are calculated based on the extracted MFCC parameters.


(4) $$diff_{m}(i)\;=\;\frac{-2m\left(i-2\right)-m\left(i-1\right)+m\left(i+1\right)+2m\left(i+2\right)}{3}$$

where *m*(·) denotes the extracted MFCC parameters, and *i* is the corresponding number of the current frame.

In the reading task, the speech length of each subject is different when reading the same text. To normalize the speech length and extract the frequency characteristics of the whole speech, the average of *m* and the *diff*_*m*_ to each mel filter are calculated. The averages of *m* and *diff*_*m*_ represent the static and dynamic average frequencies perceived by the human ear under 12 non-linear scales, respectively, which reflect the overall auditory perception frequency level of speech.

The coefficient of variation is a relative statistic used to measure the degree of dispersion of the sequence (Tian, 2010). To obtain the dispersion degree of the frequency series under 12 non-linear scales of the human ear, the coefficients of variation in the frame means of the MFCC parameters and the second-order differential MFCC parameters are calculated, which is the AFVC extracted in this work.


(5) $$AFVC=\frac{{\sum {}_{k=1}^{12}\left(\frac{\sum {}_{i=1}^{N}m_{k}\left(i\right)}{N}-mean_{ps}\right)}^{2}+\sum {}_{k=1}^{12}{\left(\frac{\sum {}_{i=1}^{N}diff_{mk}\left(i\right)}{N}-mean_{ps}\right)}^{2}}{24\;\cdot \;mean_{ps}}$$

(6) $$mean_{ps}=\frac{\sum {}_{k=1}^{12}\left(\frac{\sum {}_{i=1}^{N}m_{k}\left(i\right)}{N}\right)+\sum {}_{k=1}^{12}\left(\frac{\sum {}_{i=1}^{N}diff_{mk}\left(i\right)}{N}\right)}{24}$$

where *mean*_*ps*_ denotes the mean value of the parameter sequence, *N* is the number of frames of the current speech, 24 is the length of the parameter sequence (the number of mel filters is 12), *m*_*k*_(·) represents the extracted MFCC parameters of the *k*th Mel filter, *diff*_*mk*_(·) is the extracted second-order differential MFCC parameters of the *k*th mel filter, and *i* are defined the same as Formula 4.

The AFVC represents the dispersion degree of the MFCC parameters and the second-order differential MFCC parameters corresponding to each mel filter, which reflects the overall auditory pitch variation in speech.

#### The Proposed Head-Movement and Reading-Fluency-Related Features From the Video Modality

Schizophrenic patients show defects in head movement and reading fluency in reading tasks. Research has reported that because of impaired motor perception and speed discrimination ability, schizophrenic patients have abnormal movement in reading tasks (Whitford et al., 2018). Due to abnormalities in lexical-semantic processing (Bora et al., 2019) and grammatical ability (Tan and Rossell, 2019), the reading fluency of schizophrenic patients is also defective (Whitford et al., 2018; Mitelman et al., 2021).

Based on the above difference, the head-movement-related features and RRFs are proposed from the video modality in this work and are introduced in subsections The Proposed Head-Movement-Related Features Based on the Mapping From Three-Dimensional Head Movement to two-Dimensional Images and The Proposed Reading-Fluency Related Features Based on the Information Mined by the Transfer Learning Network From the Quantified Mouth Movement, respectively.

##### The Proposed Head-Movement-Related Features Based on the Mapping From Three-Dimensional Head Movement to Two-Dimensional Images

Clinical studies show that schizophrenia produces more unconscious and unnecessary head movements in cognitive tasks (Yoo et al., 2005). More head movements in reading tasks are associated with repeated reading (Fernández et al., 2016) and negative symptoms (Dickinson and Coursey, 2002; Chakraborty et al., 2017) in patients with schizophrenia. To quantify the subjects' head movement during the text reading task, the head-movement-related features are extracted from the video modality.

**Figure 3:**
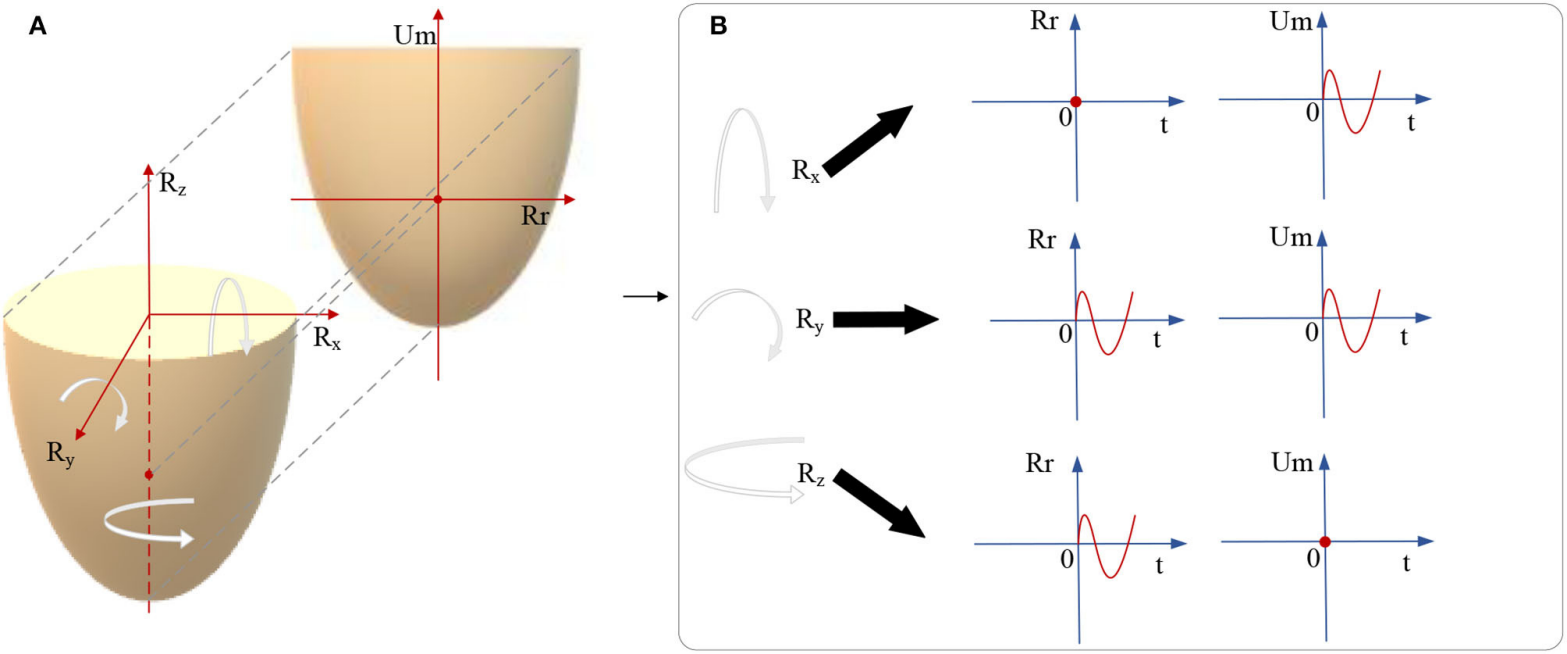
The diagram of the mapping from three head movement rotation dimensions to the dimensions of Rr and Um. **(A)** The diagram of 3D head movement mapping to 2D Rr-Um plane. **(B)** The mapping from *R*_*x*_, *R*_*y*_, and *R*_*z*_ to Rr and Um.

In the video modality, the repeated reading and unconscious head movement caused by negative symptoms of schizophrenic patients are reflected in two mutually perpendicular dimensions of the image [repeated reading dimension (Rr) and unconscious movement dimension (Um)]. Head movement has three rotational dimensions *R*_*x*_, *R*_*y*_, and *R*_*z*_. Each rotational dimension can be mapped by orthogonal decomposition to the components in the dimensions of Rr and Um (as shown in Figure 3), which achieves mapping from three-dimensional head movement to two-dimensional images.

**Figure 4:**
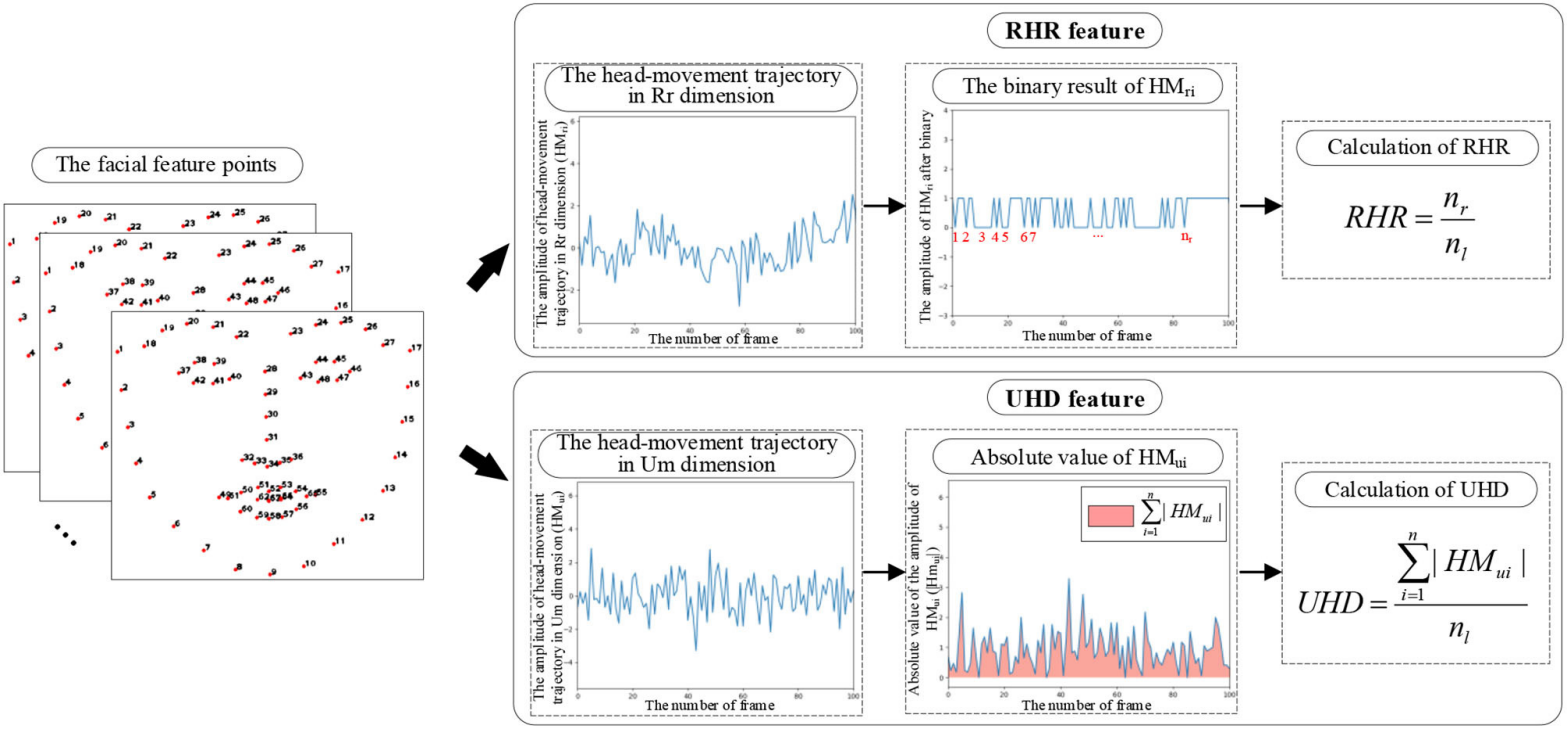
Calculation of the head-movement related features.

To quantify the subject's head movement in the two-dimensional video, the head-movement trajectories of subjects under the reading task are extracted by using the facial feature points and frame difference method. In this work, repeated-reading head-rotation ratio (RHR) and unconscious head-movement degree (UHD) are proposed based on the extracted head-movement trajectories (as shown in Figure 4), which reflect the spontaneous head movement caused by repeated reading and unconscious movement.

**A. Extraction of the head-movement trajectory**

Facial feature points at the edge of the head can reflect the position of the head. In this work, the facial feature points are extracted by using the proposed regression approach for face alignment (Ren et al., 2014). The 1–17 points of the extracted facial feature points are used in this work to determine the position of the head contour.

Calculate the abscissa and ordinate coordinate difference of the position of 1–17 points between two adjacent frames, which reflect the head movement in the dimensions of Rr and Um, respectively. Their mean value is taken as the overall frame difference of the current two adjacent frames. The calculation formulas of the head-movement trajectories in the dimensions of Rr (*HM*_*r*_) and Um (*HM*_*u*_) are as follows:


(7) $$HM_{ri}\;=\;\frac{\sum {}_{n=1}^{17}\left(x_{n_{i+1}}-x_{n_{i}}\right)}{17}$$

(8) $$HM_{ui}\;=\;\frac{\sum {}_{n=1}^{17}(y_{n_{i+1}}-y_{n_{i}})}{17}$$

where *HM*_*ri*_ and *HM*_*ui*_ represent the frame difference of frame *i* and frame *i*+1 in the dimensions of Rr and Um, respectively. (*x*_*n*_*i*__, *y*_*n*_*i*__) and (*x*_*n*_*i*+1__, *y*_*n*_*i*+1__) denote the coordinates of the *n*th point in frame *i* and frame *i*+1, respectively.

The above calculation is performed on the whole video sequence, and the obtained frame difference sequences are the head-movement trajectories in the dimensions of Rr (*HM*_*r*_) and Um (*HM*_*u*_) extracted in this work.

**B. Extraction of the head-movement-related features**

In this work, two head-movement-related features (RHR and UHD) are extracted to quantify the head movement in the Rr and Um dimensions of the two-dimensional image in the reading task.

**(1) The proposed RHR feature**

Research shows that schizophrenic patients are more prone to repeated reading in reading tasks (Fernández et al., 2016). The process of repeated reading is accompanied by head rotation, which is shown as more head movements in the horizontal direction. In addition to the necessary head rotations on a new line, the unnecessary head rotations can reflect the degree of repeated readings.

In this work, the RHR is proposed from the extracted head-movement trajectory in the Rr dimension, which quantifies the unnecessary head rotations caused by repeated reading in the reading task.

**Step 1:** Binarization of the head-movement trajectory in the Rr dimension

To extract the number of head rotations from the head-movement trajectory in the Rr dimension, the head-movement trajectory in the Rr dimension is transformed into a binary sequence. The transforming rule is that the right motion is set to 1, and the left motion is set to 0. The binarized head-movement trajectory in the Rr dimension only contains the direction information of the head in the Rr dimension, which directly reflects the subject's head rotation.

**Step 2:** Calculation of RHR

Considering the difference in the length of four texts, the ratio of the extracted number of head rotations to the number of text lines is taken as the RHR. RHR normalizes the effect of the number of text lines on the head rotation.


(9) $$\begin{array}{r} RHR\;=\;\frac{n_{r}}{n_{l}} \end{array}$$

where *n*_*r*_ is the extracted number of head rotations and *n*_*l*_ is the number of text lines.

As the influence of text length is normalized, RHR represents the unnecessary head-rotation of the subject in the reading task. Unnecessary head-rotation reflects the number of repeated readings, and hence, the RHR reflects the degree of repeated reading of the subject in the reading task.

**(2) The proposed UHD feature**

The unconscious head movement in schizophrenia caused by negative symptoms can be reflected in the Um dimension of the images. When the text position is fixed, the necessary vertical head-movement amplitude caused by reading is similar. For different subjects, the difference in the sum of the amplitude of vertical head-movement reflects the difference in the degree of unconscious head movement.

In this work, the UHD is proposed to quantify the degree of head movement in the Um dimension of the subjects during the reading task, which reflects the amount of unconscious head movement during the reading task.

To normalize the effect of the number of text lines on head movement in the Um dimension, the ratio of the sum of the amplitude of head movement in the Um dimension to the number of text lines is taken as the UHD.


(10) $$UHD\;=\;\frac{\sum {}_{i=1}^{n}|HM_{ui}|}{n_{l}}$$

where *HM*_*ui*_ denotes the *i*th value of the head-movement trajectory in the Um dimension, *n* is the length of the head-movement trajectory in the Um dimension, and *n*_*l*_ is the number of text lines.

##### The Proposed Reading-Fluency-Related Features Based on the Information Mined by the Transfer Learning Network From the Quantified Mouth Movement

Research shows that schizophrenic patients have defects in reading comprehension and phonological processing in reading tasks (Arnott et al., 2011). Reading comprehension and phonological processing have an impact on reading fluency, especially for schizophrenic patients who use Chinese (Wang et al., 2015). Clinical studies have proven that schizophrenic patients tend to show poor reading fluency due to defects in reading ability (Whitford et al., 2018; Mitelman et al., 2021).

In this work, mouth movement is quantified to reflect the reading situation of the subjects. Based on the quantified mouth movement and transfer learning, RRF is proposed in this work.

**A. Quantitative characterization of mouth movement**

To extract the RRFs based on mouth movement, the subjects' mouth movement during the reading task needs to be quantified. In this work, the mouth aspect ratio sequence is extracted based on the facial feature points. To normalize the obtained mouth aspect ratio sequence and extract the features related to reading fluency, the mouth aspect ratio sequence is mapped into the graph from the time domain and frequency domain. The specific steps are as follows:

**Step 1:** Extraction of the mouth aspect ratio sequence

The facial feature points of mouth contour can be used to calculate the mouth aspect ratio (Ren et al., 2014). In this work, the distance between points 49 and 55 represents the length of the mouth. The distance between points 52 and 58 represents the width of the mouth. The ratio of the mouth width to length is the mouth aspect ratio (MAR).


(11) $$\begin{array}{r} MAR\;=\;\frac{\sqrt{{\left(x_{P_{52}}-x_{P_{58}}\right)}^{2}+{\left(y_{P_{52}}-y_{P_{58}}\right)}^{2}}}{\sqrt{{\left(x_{P_{49}}-x_{P_{55}}\right)}^{2}+{\left(y_{P_{49}}-y_{P_{55}}\right)}^{2}}} \end{array}$$

where (*x*_*P*_49__, *y*_*P*_49__), (*x*_*P*_52__, *y*_*P*_52__), (*x*_*P*_55__, *y*_*P*_55__) and (*x*_*P*_58__, *y*_*P*_58__) represent the coordinates of points 49, 52, 55, and 58, respectively.

The mouth aspect ratio is calculated according to the frame of the video sequence, and the obtained sequence is the mouth aspect ratio sequence.

**Step 2:** Mapping from the time domain and frequency domain

**Time domain:** The length of each subject's extracted mouth aspect ratio sequence is different because of the different reading time of each subject when reading the same text. To normalize the length of the mouth aspect ratio sequence and avoid the loss of sequence information in the time domain, each sequence is mapped to an RGB image according to its sequence value from the time domain.

To reflect the time-varying amplitude of the mouth aspect ratio sequence from the map, the pixel coding rules are designed as follows: the pixel values of the R channel in the *i*th row and column are encoded as 200 times the *i*th value of the sequence, and the pixel values of the G and B channels are encoded as 0.

The proposed mouth movement time-domain map contains the information of all elements in the sequence, which reflects the change in the mouth aspect ratio sequence with time.

**Frequency domain:** The mouth aspect ratio sequence contains the mouth movement frequency information related to reading fluency, which can be extracted from the frequency domain. To supplement the frequency domain information, a mouth movement three-dimensional spectrogram is proposed in this work.

To extract the three-dimensional spectrogram of mouth movement, the mouth aspect ratio sequence is processed by short-time segmentation, with 3s as the segment length and 1s as the segmentation interval. For each short-time mouth aspect ratio sequence, the short-time Fourier transform (STFT) is used to obtain its corresponding frequency amplitude.

The proposed three-dimensional spectrogram reflects the time-varying relationship of the short-time frequency spectrum of the time series, which supplements the frequency information contained in the mouth aspect ratio sequence.

**B. Extraction of the RRFs**

To make use of the ability of deep learning to mine the image information and avoid the impact of the small-scale database on accuracy, transfer learning is used to build the feature extraction network in this work.

**Figure 5:**
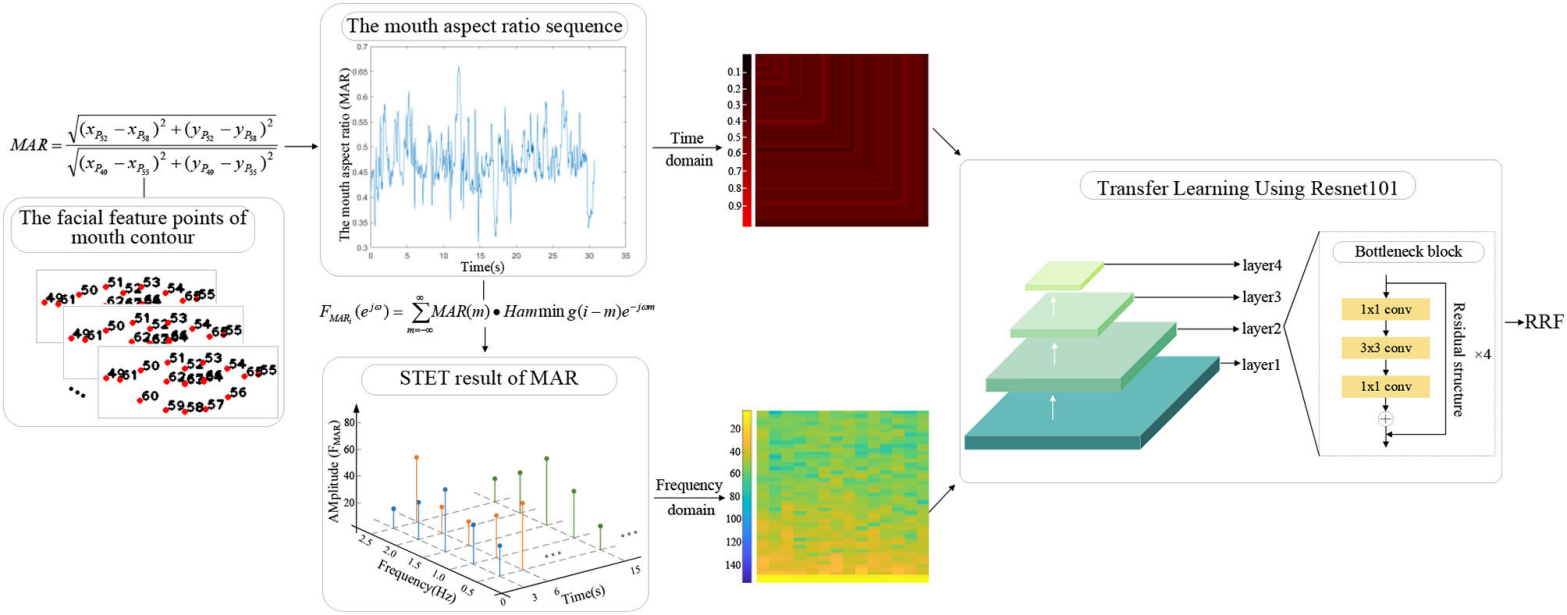
Extraction of the reading-fluency-related features.

The convolution structure in ResNet101 can mine image information. The introduction of the residual structure in ResNet101 ensures the depth of the network, and the design of the bottleneck module assures the stable improvement of network performance layer by layer (Deng et al., 2009; He et al., 2016). The deep network structure and stable improved performance enable ResNet101 to mine depth information of the proposed mouth movement time-domain map and three-dimensional spectrogram through multilayer convolution (as shown in Figure 5).

For the ResNet101 pretrained model, the convolution layer is frozen to extract features, and the fully connected layer is reset to binary classification. The inputs of the network are the proposed mouth movement time-domain map and the proposed three-dimensional spectrogram. RRF is the feature extracted from the fully connected layers of the transfer learning model, which contains both the time-varying amplitude information and the mouth movement frequency information.

RRF contains the information mined by the transfer learning network from the mouth movement time-domain map and the three-dimensional spectrogram, which reflects the reading-fluency situation according to the mouth movement.

## Results and Analysis

To automatically diagnose schizophrenia, an automatic schizophrenia detection algorithm is proposed from the speech and video modality based on the abnormalities of schizophrenic patients in the reading task in this work. In the speech modality, the acoustic model of speech emotional flatness in schizophrenia is established to reflect the emotional expression defects in schizophrenia. In the video modality, the relevant features are extracted from the head-movement and reading fluency aspects. In the head-movement aspect, RHR and UHD are extracted to quantify the head movement in the repeated reading dimension and unconscious movement dimension, respectively. In the reading fluency aspect, the transfer learning method is used to mine the features (RRF) in the proposed mouth movement time-domain map and the three-dimensional spectrogram. In this section, the results of the automatic schizophrenia detection algorithm based on multimodality in the reading task are illustrated in subsection Results of the Automatic Schizophrenia Detection Algorithm Based on Multimodality in the Reading Task. The results of the acoustic model of speech emotional flatness in schizophrenia and the proposed features in the video modality are displayed and analyzed in subsections Results of the Proposed Acoustic Model of Speech Emotional Flatness in Schizophrenia and Results of the Proposed Features in Video Modality, respectively.

### Results of the Automatic Schizophrenia Detection Algorithm Based on Multimodality in the Reading Task

In this work, the proposed automatic schizophrenic detection algorithm uses the proposed feature set from multimodality to realize the automatic classification of schizophrenia and normal controls. The feature set consists of the features proposed from the speech modality (AFVC, PIVD) and the video modality (RHR, UHD, RRF). The classification results of the automatic schizophrenia detection algorithm combined with SVM and RF by using 10-fold cross-validation are shown in Table 1. To reduce the impact of occasionality caused by randomly dividing the original samples in cross-validation, 10-fold cross-validation is conducted 5 times, and the mean value is taken as the final result.

**Table 1:** Results of the proposed automatic schizophrenic detection algorithm.

**Modality**	**Feature**	**Classifier**	**Acc. (%)**	**Spec. (%)**	**Sens. (%)**	**AUC (%)**
Speech	AFVC+PIVD	SVM	94.38	97.00	91.75	97.13
		RF	96.50	97.25	95.75	99.72
Video	RHR+UHD+RRF	SVM	86.63	83.75	89.50	95.13
		RF	81.25	80.00	82.50	92.19
Speech+Video	AFVC+PIVD+RHR+UHD+RRF	SVM	96.25	97.00	95.50	99.66
		RF	97.50	98.50	96.50	99.75

In this experiment, accuracy (Acc.), specificity (Spec.), sensitivity (Sens.), and AUC are used to evaluate the classification performance of the proposed features. The accuracy is the proportion of samples with correct classification in the total samples. The specificity is the proportion of correctly classified normal controls in all normal controls, which reflects the diagnostic rate of the proposed classification method. The sensitivity is the proportion of correctly classified schizophrenic patients in all patients, reflecting the probability of no missed diagnosis in the proposed classification method. AUC is the area under the ROC curve, which represents the authenticity of the proposed classification method.

In the feature set, AFVC and PIVD reflect the emotional flatness of schizophrenic speech, RHR and UHD manifest spontaneous head movement in the Rr and Um dimensions, and RRF embodies the defects in the reading fluency of schizophrenic patients. The proposed feature set combines the information in speech and video modalities, which reflects the reading abnormalities of schizophrenic patients. As shown in Table 1, the classification accuracy of the proposed algorithm in speech modality is 94.38 and 96.50%, and AUC is 97.13 and 99.72%. The classification accuracy of the proposed algorithm in video modality is 86.63 and 81.25%, and AUC is 95.13 and 92.19%. By combining the features proposed from multimodality, the classification accuracy of the proposed automatic schizophrenic detection algorithm is 96.25 and 97.50%, and the AUC is 99.66 and 99.75%. The comparison results between multimodality and single modality indicate that the combination of speech and video modalities improves the classification performance.

Previous clinical research has reported that schizophrenic patients have reading defects (Revheim et al., 2014; Whitford et al., 2018), which can be reflected in the abnormal speech (Meyer et al., 2021; Tan et al., 2021), head movement (Yoo et al., 2005; Abbas et al., 2021), and reading fluency (Whitford et al., 2018; Mitelman et al., 2021) of schizophrenia. In this work, to reflect the reading defects of patients with schizophrenia, the proposed automatic schizophrenia detection algorithm integrates the defects in the above three aspects from the speech and video modalities. It can be seen from the experimental results that the fused features proposed from multimodality achieved good classification performance for schizophrenia.

### Results of the Proposed Acoustic Model of Speech Emotional Flatness in Schizophrenia

PIVD and AFVC are the proposed features from the established acoustic model of speech emotional flatness in schizophrenia, which reflect the emotional expression of speech from the perspective of speech production and perception. Due to the symptoms of emotional flattening, the emotional expression ability of schizophrenic patients is defective, which manifests as the decreased speech intensity and pitch variations from the perspective of speech production and perception, respectively. The results of the proposed acoustic model of speech emotional flatness in schizophrenia are shown in Table 2.

**Table 2:** Results of the proposed acoustic model of speech emotional flatness in schizophrenia.

**Feature**	**Classifier**	**Acc. (%)**	**Spec. (%)**	**Sens. (%)**	**AUC (%)**
PIVD	SVM	93.50	96.50	90.50	96.97
	RF	93.13	92.00	94.25	99.03
AFVC	SVM	92.88	90.00	95.75	95.88
	RF	90.38	91.25	89.50	98.59

As shown in Table 2, the classification accuracy of PIVD and AFVC is more than 90%. The accuracy of PIVD under the two classifiers is 93.50 and 93.13%, and the AUC is 96.97 and 99.03%, respectively. The accuracy of AFVC under the two classifiers is 92.88 and 90.38%, and the AUC is 95.88 and 98.59%, respectively.

**Figure 6:**
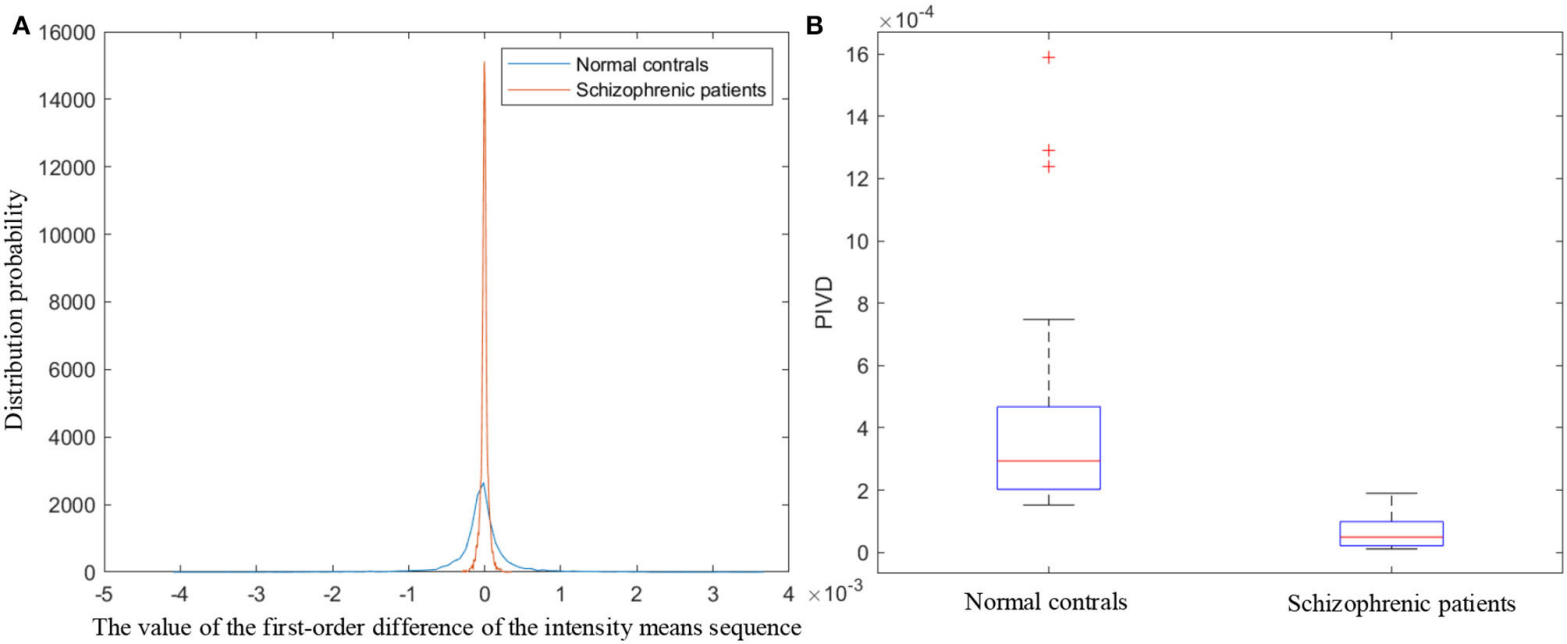
**(A)** The probability density function curves of the first-order difference of the intensity means sequence. **(B)** Boxplot of PIVD.

The proposed PIVD quantifies the speech intensity variations by calculating the standard deviation of the first-order difference of the frame means of the overall speech intensity (*diff*_*IS*_). Figure 6A illustrates the probability density function curves of *diff*_*IS*_ of normal controls and schizophrenic patients. As shown in Figure 6A, the distribution of *diff*_*IS*_ of schizophrenic speech is smaller and more concentrated. *diff*_*IS*_ represents the intensity variation in the speech over time. The smaller and more concentrated distribution of *diff*_*IS*_ agrees with the more monotonous speech intensity variation in schizophrenia caused by emotional flattening (Compton et al., 2018; He et al., 2021).

The boxplot of PIVD is shown in Figure 6B. In each box, the red line indicates the median, and the bottom and top edges of the box indicate the 25th and 75th percentiles, respectively. The outliers are plotted individually using the ‘+' symbol. As the boxplot shown in Figure 6B, the overall PIVD of normal controls is larger than that of schizophrenic patients, which indicates that the diversity of the speech intensity variations in schizophrenic patients is lower than that in normal controls.

**Figure 7:**
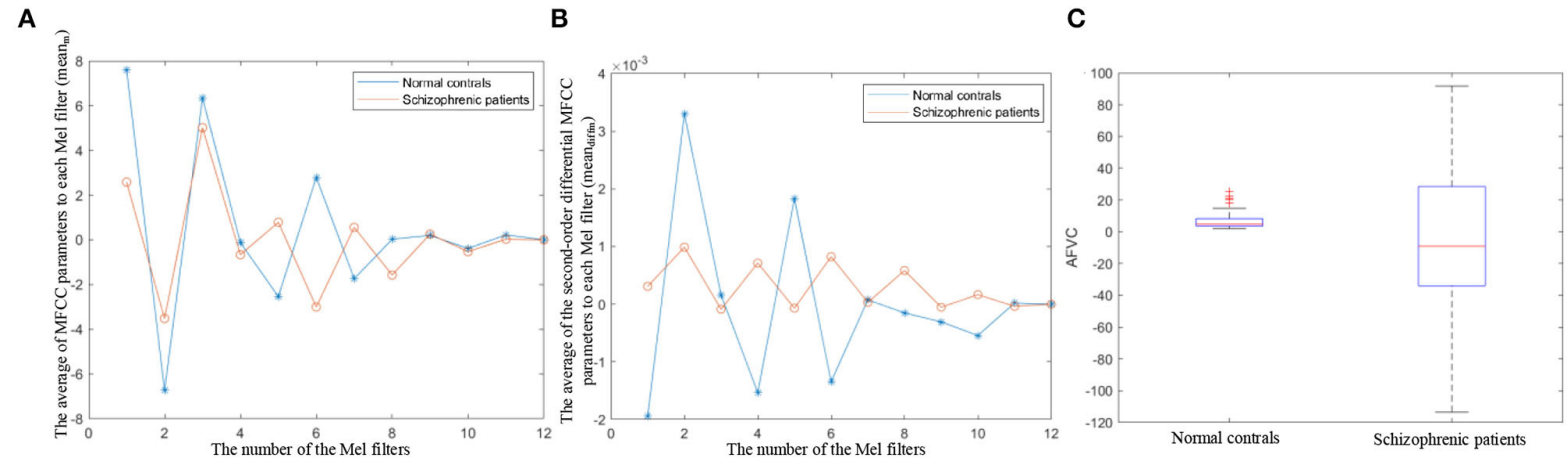
**(A)** The distribution of the average of MFCC parameters to each mel filter. **(B)** The distribution of the average of the second-order differential MFCC parameters to each mel filter. **(C)** Boxplot of AFVC.

MFCC is the cepstrum parameters extracted in the mel scale frequency domain, which reflect the speech frequency from the perspective of hearing (Ali et al., 2021). The variation degree in MFCC reflects auditory pitch variation. The proposed AFVC quantifies the variation degree of MFCC by calculating the dispersion of the average of MFCC parameters (*mean*_*m*_) and the average of second-order differential MFCC parameters to each mel filter (*mean*_*diffm*_). Figures 7A,B illustrate the specific distributions of *mean*_*m*_ and *mean*_*diffm*_, respectively.

As shown in Figures 7A,B, the dispersion of the *mean*_*m*_ and *mean*_*diffm*_ of schizophrenic speech is lower than that of normal speech. *mean*_*m*_ and *mean*_*diffm*_ represent the static and dynamic average frequencies perceived by the human ear under 12 non-linear scales. The lower dispersion of the *mean*_*m*_, *mean*_*diffm*_ reflects lower auditory pitch variation, which is in line with the lower expression variation in schizophrenia from speech perception (Leitman et al., 2010; Henry et al., 2015).

As the boxplot shows in Figure 7C, the median AFVC of normal controls is larger than that of schizophrenic patients, and the AFVC distribution of the normal controls is more concentrated. It can be seen from the overall statistical results that when reading the fixed text, the speech pitch variation in the normal controls is more obvious, and the variation degree is similar among different normal subjects. On the contrary, the overall speech pitch variations of schizophrenic patients are less, and the differences between different patients are greater.

### Results of the Proposed Features in Video Modality

The proposed features in the video modality are composed of head-movement-related features and RRFs. The head-movement-related features are proposed based on the mapping from three-dimensional head movement to two-dimensional images. The RRFs are proposed based on the information mined by transfer learning from the time and frequency domains of the mouth movement sequences. The results of the proposed features in video modality are shown in Table 3. The results of head-movement-related features and RRFs are detailed in subsections Results of the Proposed Head-Movement-Related Features and The Reading-Fluency-Related Features (RRF), respectively.

**Table 3:** Results of the proposed features in video modality.

**Feature**	**Classifier**	**Acc. (%)**	**Spec. (%)**	**Sens. (%)**	**AUC (%)**
RHR	SVM	83.38	92.00	74.75	87.13
	RF	73.38	74.25	72.50	88.28
UHD	SVM	80.88	91.00	70.75	82.88
	RF	73.63	74.50	72.75	86.53
RRF	SVM	83.63	85.25	82.00	90.63
	RF	79.50	79.25	79.75	91.00

#### Results of the Proposed Head-Movement-Related Features

RHR and UHD are the proposed head-movement-related features, which reflect the spontaneous head-movement caused by repeated reading and unconscious movement. Clinical studies have shown that schizophrenic patients show more repeated reading (Fernández et al., 2016) and more limb movement (Chakraborty et al., 2017) in reading tasks. The more repeated reading and limb movement cause schizophrenic patients to have more head movement in the proposed dimensions of Rr and Um than normal controls.

In the video modality, RHR and UHD reflect the head movement of the subjects in the dimensions of Rr and Um. As shown in Table 3, the accuracy of RHR under the two classifiers is 83.38 and 73.38%, and the AUC is 87.13 and 88.28%, respectively. The accuracy of UHD under the two classifiers is 80.88 and 73.63%, and the AUC is 82.88 and 86.53%, respectively.

**Figure 8:**
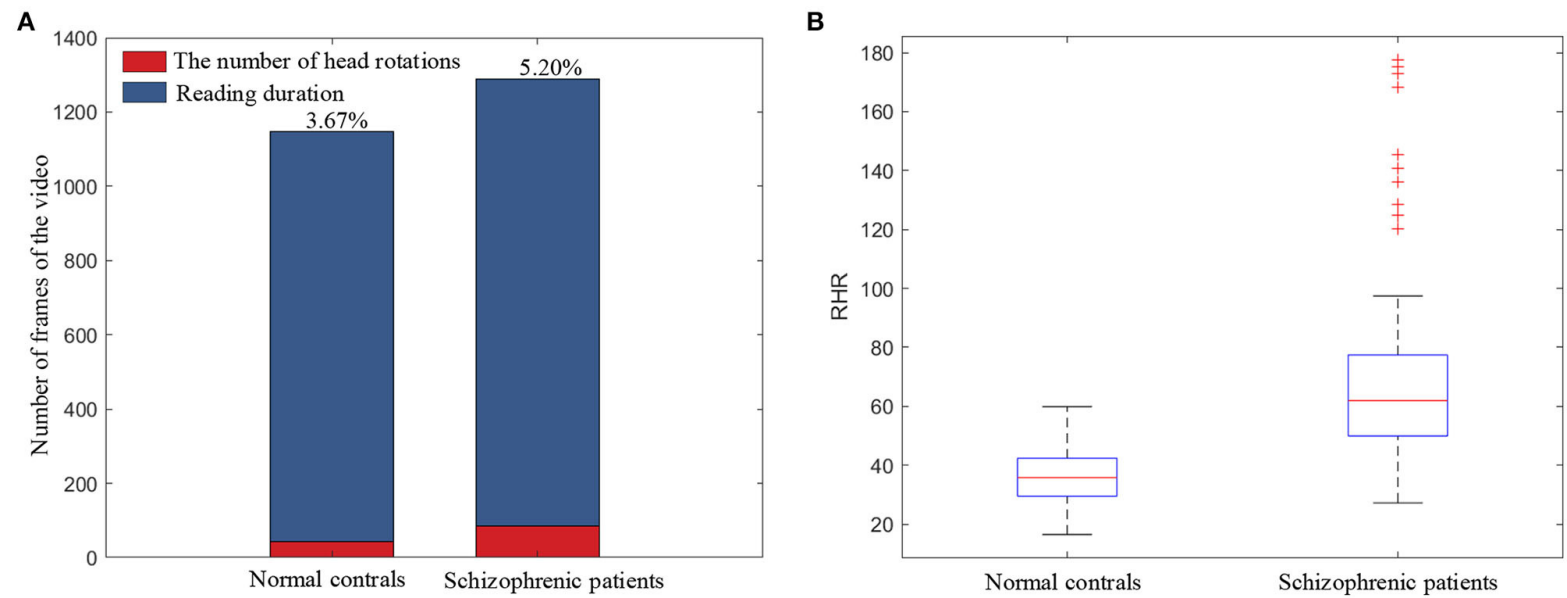
**(A)** The ratio of head rotations to the reading duration. **(B)** Boxplot of RHR.

RHR is the ratio of the head-rotation number to the text line number, which represents the subjects' head-movement degree in the Rr dimension in the reading task. The number of head rotations is extracted from the binarization of the head-movement trajectory in the Rr dimension (*HM*_*r*_). Figure 8A illustrates the ratio of head rotations to the reading duration of schizophrenic patients and normal controls. As shown in Figure 8A, schizophrenic patients need a longer time and have more head rotations when reading the same text as normal controls. The longer time and more head rotations are consistent with the larger number of repeated readings in schizophrenic patients in previous clinical research (Fernández et al., 2016).

The boxplot of RHR is shown in Figure 8B. As shown in the boxplot, the overall RHR of schizophrenic patients is larger than that of normal controls. The overall statistical results in the boxplot prove the universality of the more head movement in the Rr dimension caused by repeated reading of schizophrenic patients.

**Figure 9:**
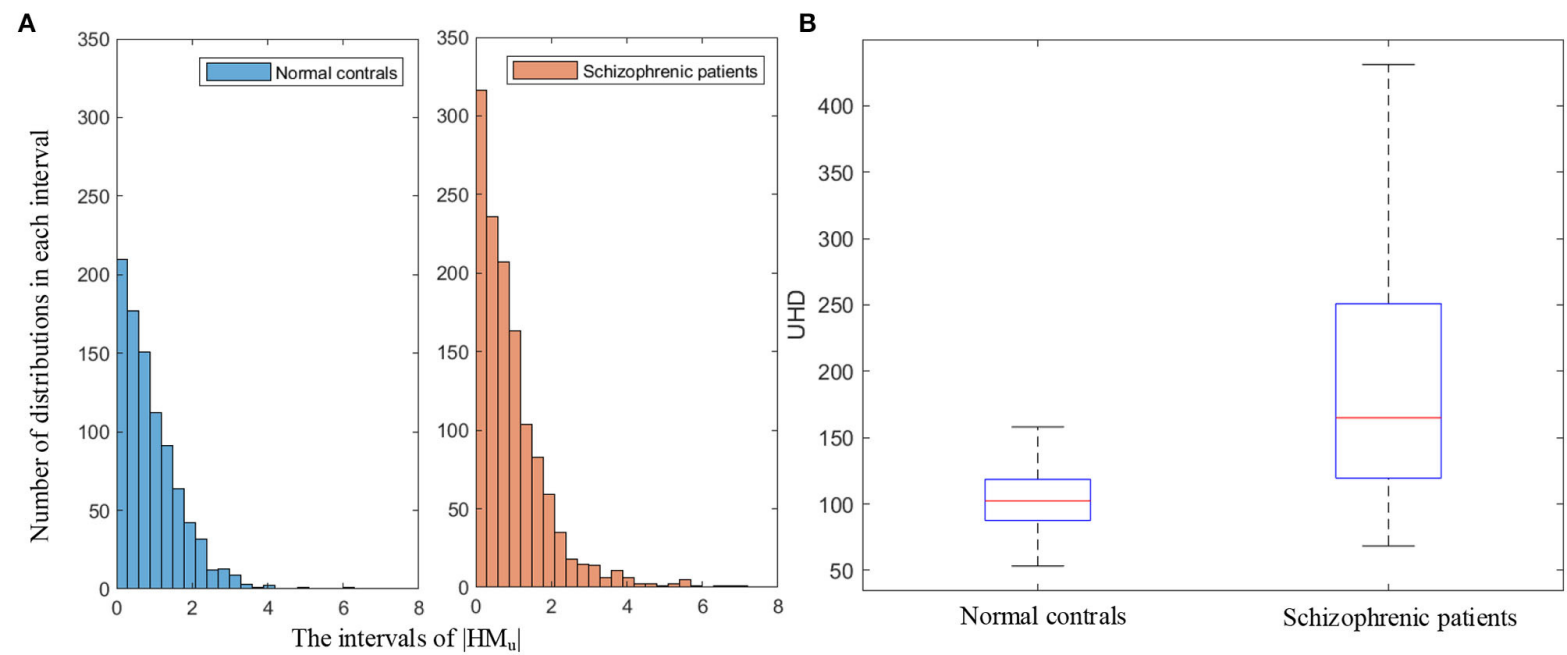
**(A)** Histograms of the amplitude of head movement in the Um dimension (|*HM*_*u*_|). **(B)** Boxplot of UHD.

UHD is the ratio of the sum of the amplitude of head movement in the Um dimension to the number of text lines, which quantifies the subjects' unconscious head movement. Figure 9A illustrates the histograms of the amplitude of head movement in the Um dimension (|*HM*_*u*_|) for normal controls and schizophrenic patients. As shown in Figure 9A, the amplitude of head movement in the Um dimension of schizophrenic patients has a wider distribution range and a larger number of distributions in each interval. It can be seen in the statistical results of the histograms that compared with the normal controls, schizophrenic patients have greater amplitude and more head movement in the Um dimension, which conforms to the more unconscious head movement in schizophrenic patients in the clinic (Yoo et al., 2005).

The boxplot of UHD is shown in Figure 9B. As shown in the boxplot, the overall UHD of schizophrenic patients is larger than that of normal controls, which indicates the universality of the more head movement in the Um dimension of schizophrenic patients.

#### The Reading-Fluency-Related Features (RRF)

RRF is the feature set consisting of the proposed RRFs extracted from the subjects' mouth movement by using transfer learning. Clinical research shows that schizophrenic patients have defects in reading comprehension and phonological processing in reading tasks (Arnott et al., 2011), which lead to worse reading fluency in schizophrenic patients than in normal controls (Whitford et al., 2018; Mitelman et al., 2021). As shown in Table 3, the accuracy of RRF under the two classifiers is 83.63 and 79.50%, and the AUC is 90.63 and 91.00%, respectively.

In this experiment, the proposed mouth movement time-domain map and the three-dimensional spectrogram are used as the input of the transfer learning network, respectively. The pretrained transfer learning network extracts two features from each input, hence the proposed RRF is a feature set containing four features.

**Figure 10:**
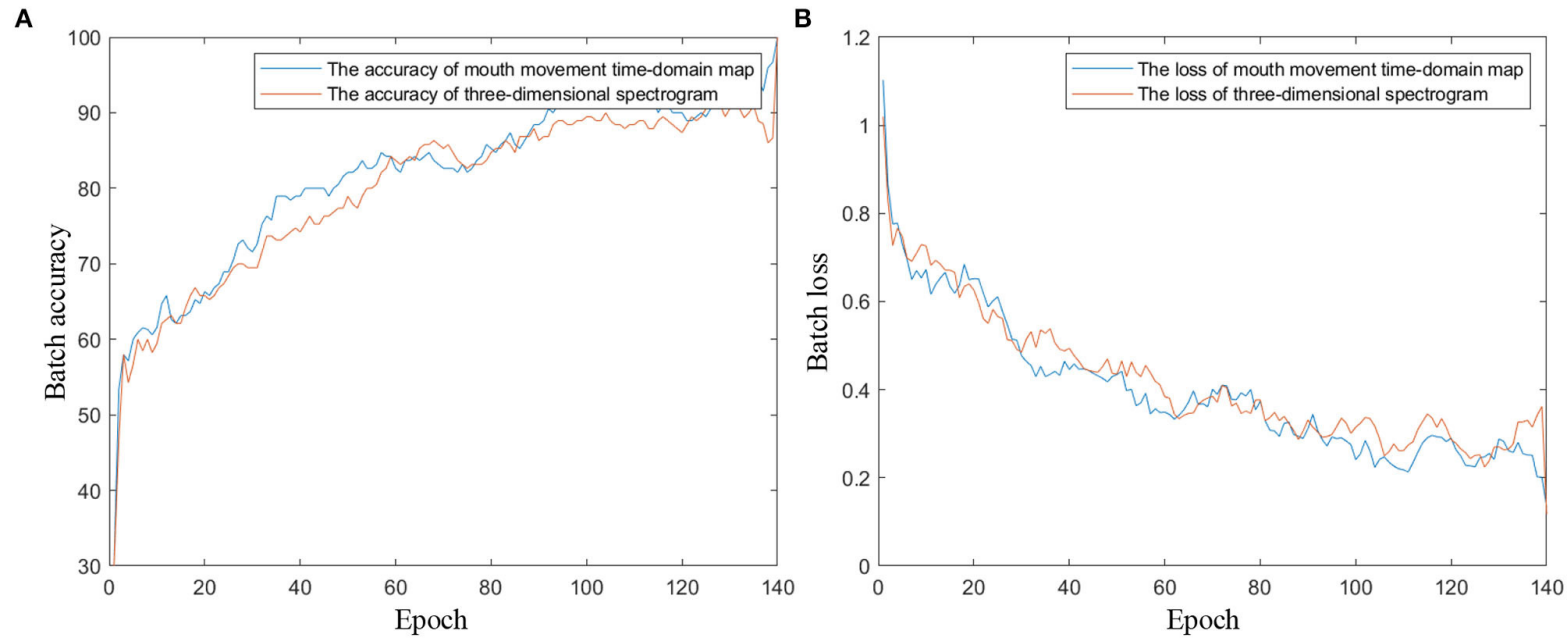
The training progress diagrams of the transfer learning network. **(A)** Training accuracy curves. **(B)** Training loss curves.

In the network training process, the mini-batch size is set to 10, the epoch number is 140, and the learning rate is 0.0001. Figure 10 shows the training progress diagrams with the proposed mouth movement time-domain map and the three-dimensional spectrogram as the input of the network, respectively. As shown in Figure 10, after 140 training epochs, the networks of the two inputs reach an accuracy rate of ~90% and a loss rate of ~0.3%. The training accuracy and loss curves indicate that the pretrained convolution layers improve the convergence speed for the network, and the image information mining ability of ResNet101 ensures the classification accuracy of the network.

**Figure 11:**
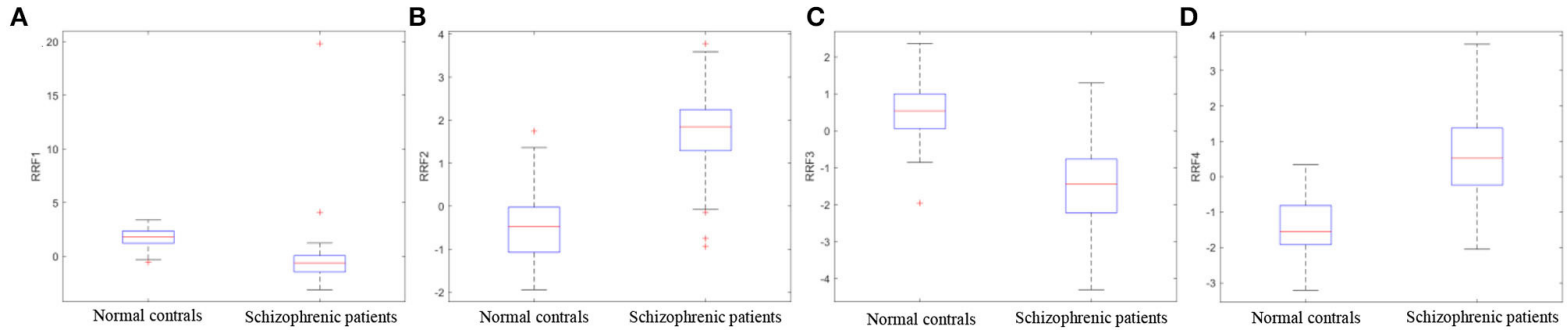
The boxplots of RRF. **(A–D)** represent the boxplot of each feature in the RRF feature set.

Figure 11 illustrates the boxplots of RRF extracted from one of the 10-fold cross-validations. As shown in the boxplots, each feature in the RRF feature set shows the differentiation between schizophrenic patients and normal controls, which certifies the defects in reading fluency in patients with schizophrenia (Whitford et al., 2018; Mitelman et al., 2021) and proves the effectiveness of RRF as an objective index for the diagnosis of schizophrenia.

### Results of the State-of-the-Art

Previous studies (Kupper et al., 2010, 2015; Chakraborty et al., 2018; Compton et al., 2018; Tahir et al., 2019) have reported defects in the speech expression and head movement of schizophrenic patients. The speech expression-related features are proposed based on the exception of speech pitch (Chakraborty et al., 2018; Compton et al., 2018) and intensity (Chakraborty et al., 2018; Tahir et al., 2019). The head-movement-related feature is the head movement amount proposed by using motion energy analysis (MEA) (Kupper et al., 2010, 2015). In this section, comparative experiments of the above features are conducted using 10-fold cross-validation. The results of the state-of-the-art are shown in Table 4.

**Table 4:** Results of the state-of-the-art.

**Feature type**	**Feature**	**Classifier**	**Acc. (%)**	**Spec. (%)**	**Sens. (%)**	**AUC (%)**
Speech expression	Mean value of pitch (Chakraborty et al., 2018)	SVM	74.38	68.75	80.00	83.59
		RF	70.63	68.75	72.50	83.28
	Standard deviation of pitch (Compton et al., 2018)	SVM	59.38	71.25	47.50	63.59
		RF	51.25	57.50	45.00	57.34
	Mean value of intensity (Tahir et al., 2019)	SVM	66.25	56.25	76.25	69.09
		RF	56.25	52.50	60.00	71.41
	Standard deviation of intensity (Chakraborty et al., 2018)	SVM	54.38	61.25	47.50	53.59
		RF	50.00	50.00	50.00	52.34
Head movement	Head movement amount extracted by using MEA (Kupper et al., 2010, 2015)	SVM	57.50	72.50	42.50	68.13
		RF	52.50	47.50	57.50	61.41

The speech expression-related features in state-of-the-art are proposed based on the basic statistical characteristics of pitch and intensity, which do not consider the variations in pitch and intensity from the perspective of the speech generation mechanism and auditory perception. As shown in Table 4, the accuracy of the speech expression-related features ranges from 50.00 to 74.38%, and the AUC range from 52.34 to 83.59%, which are lower than those of the proposed acoustic model of speech emotional flatness in schizophrenia in this work. Compared with the speech expression-related features in state-of-the-art, the proposed acoustic model of speech emotional flatness in schizophrenia in this work illustrates the emotional flattening symptoms of schizophrenia from the perspective of speech production and perception. The results of the comparative experiments prove the better classification performance for schizophrenia of the proposed acoustic model in this work.

The head-movement-related feature in state-of-the-art is the amount of head movement quantified by using MEA, which ignores the specific movement trajectory. As shown in Table 4, the accuracy of the head-movement-related features ranges from 52.50 to 57.50%, and the AUC ranges from 61.41 to 68.13%, which are lower than those of the proposed head-movement-related features (RHR and USD) in this work. Compared with the amount of head movement extracted by using MEA, the proposed head-movement-related features in this work contain the trajectory information of head movement and reflect the spontaneous head movement caused by repeated reading and unconscious movement. The results of the comparative experiments prove that the proposed head-movement-related features in this work perform better in the automatic diagnosis of schizophrenia.

## Discussion and Conclusion

In this research, a new automatic schizophrenia detection algorithm is proposed based on the abnormal performance of schizophrenic patients in the reading task. The automatic schizophrenia detection algorithm involves the features proposed from the speech and video modalities. In the speech modality, an acoustic model of speech emotional flatness in schizophrenia is established from the perspective of speech production and perception, which reflects the emotional flatness of schizophrenia. In the video modality, the head-movement-related features are proposed based on the mapping from three-dimensional head movement to two-dimensional images, which reflect the spontaneous head movement caused by repeated reading and unconscious movement. The RRFs are proposed from the mouth movement by using transfer learning, which reflects the degree of subjects' reading fluency.

To test the classification performance, experiments on the proposed automatic schizophrenia detection algorithm and the state-of-the-art are conducted. Accuracy (Acc.), specificity (Spec.), sensitivity (Sens.), and AUC are the performance evaluation indices of the experiments. Through the experiments with 10-fold cross-validation, the diagnostic accuracy of the proposed acoustic model of speech emotional flatness in schizophrenia, the head-movement related features, and the reading-fluency related features range from 94.38 to 96.50%, 73.38 to 83.38%, and 79.50 to 83.63%, respectively. The proposed automatic schizophrenia detection algorithm combines the features of the speech and video modalities, and its classification accuracy ranges from 96.25 to 97.50%. It can be seen from the experimental results that the integration of the features in multimodality shows better classification performance than those in any one modality, which combines the reading deficits of schizophrenic patients in speech, head movement, and reading fluency. The results of this work indicated that the proposed automatic detection algorithm can be used for the assisted auxiliary diagnosis of schizophrenia.

## Data Availability Statement

The datasets presented in this article are not readily available because they contain participants identifiable facial images and must be approved by the Psychiatry Department of the Mental Health Center, Sichuan University. Requests to access the datasets should be directed to LH, ling.he@scu.edu.cn.

## Ethics Statement

The studies involving human participants were reviewed and approved by the Psychiatry Department of the Mental Health Center, Sichuan University. Written informed consent to participate in this study was provided by the participants' legal guardian/next of kin. Written informed consent was obtained from the individual(s) for the publication of any potentially identifiable images or data included in this article.

## Author Contributions

All authors listed have made a substantial, direct, and intellectual contribution to the work and approved it for publication.

## Funding

This work was supported by grants from the National Natural Science Foundation of China (No. 81901389), Innovation Spark Project of Sichuan University (No. 2018SCUH0002), and Sichuan University-Yibin School-City Strategic Cooperation Special Fund Project (No. 2020CDYB-27).

## Conflict of Interest

The authors declare that the research was conducted in the absence of any commercial or financial relationships that could be construed as a potential conflict of interest.

## Publisher's Note

All claims expressed in this article are solely those of the authors and do not necessarily represent those of their affiliated organizations, or those of the publisher, the editors and the reviewers. Any product that may be evaluated in this article, or claim that may be made by its manufacturer, is not guaranteed or endorsed by the publisher.
